# Signaling Pathways in Cancer: Therapeutic Targets, Combinatorial Treatments, and New Developments

**DOI:** 10.3390/cells10030659

**Published:** 2021-03-16

**Authors:** Hon Yan Kelvin Yip, Antonella Papa

**Affiliations:** Cancer Program, Monash Biomedicine Discovery Institute and Department of Biochemistry and Molecular Biology, Monash University, Melbourne, VIC 3800, Australia; kelvin.yip@monash.edu

**Keywords:** signaling pathways, targeted therapies, combinatorial treatments, oncogenes and tumor suppressors, cancer resistance, RTK, PROTACS

## Abstract

Molecular alterations in cancer genes and associated signaling pathways are used to inform new treatments for precision medicine in cancer. Small molecule inhibitors and monoclonal antibodies directed at relevant cancer-related proteins have been instrumental in delivering successful treatments of some blood malignancies (e.g., imatinib with chronic myelogenous leukemia (CML)) and solid tumors (e.g., tamoxifen with ER positive breast cancer and trastuzumab for HER2-positive breast cancer). However, inherent limitations such as drug toxicity, as well as acquisition of de novo or acquired mechanisms of resistance, still cause treatment failure. Here we provide an up-to-date review of the successes and limitations of current targeted therapies for cancer treatment and highlight how recent technological advances have provided a new level of understanding of the molecular complexity underpinning resistance to cancer therapies. We also raise three basic questions concerning cancer drug discovery based on molecular markers and alterations of selected signaling pathways, and further discuss how combination therapies may become the preferable approach over monotherapy for cancer treatments. Finally, we consider novel therapeutic developments that may complement drug delivery and significantly improve clinical response and outcomes of cancer patients.

## 1. Introduction

Cancer is a disease characterized by the presence of cells undergoing continuous cell divisions in a rapid and uncontrolled manner. While in the early days surgery and chemotherapies were the only means available to treat tumors, the discovery of oncogenes and tumor suppressor genes has advanced the notion that single proteins can be targeted pharmacologically for cancer therapy. Recent advances in next-generation sequencing and multi-omics analyses have further revealed how signaling pathways are intimately intertwined to form complex circuitries within cells that, if fully mapped, can be exploited for more accurate targeted therapies.

In this review, we discuss some of the recent advances in cancer drug discovery and provide examples of signaling-based cancer therapies. We further discuss how synthetic lethality can be utilized to perturb “undruggable” targets, i.e., difficult to target proteins, and also to treat drug-resistant cancers. A large number of signaling nodes and molecular hubs have been implicated in the occurrence of cancer, and many of these, such as receptor tyrosine kinases (RTKs) and downstream signaling pathways, are current targets of drugs approved by multiple regulatory authorities (e.g., the U.S. Food and Drug Administration, FDA; European Medicines Agency, EMA).

## 2. What Oncogenic Perturbations Are Currently Known and Available as Anti-Cancer Targets?

### 2.1. Historical Perspective of Chemotherapy

Given the uncontrolled proliferative capacity distinctive of benign tumor and malignant cancer cells, local surgical resection and radiotherapy appeared to be a logical and effective way to treat tumors in the early days. However, tumors and cancers are more than just a mass of rapidly proliferating cells; they are indeed a highly complex disease that requires many elements to thrive, including a compromised microenvironment surrounding the primary tumor, as well as an adapted systemic response whereby a permissive immune system [1,2] and microbiome [3] allow the tumor to evolve. Additional factors such as intra-tumor heterogeneity and clonal evolution further challenge our appreciation of the complexity of tumor biology and dangerously undermine our ability to choose the most effective treatment at the point of care.

Modern chemotherapy began in the 1970s with the use of antimitotic drugs such as cyclophosphamide and chlorambucil, which were prescribed for the treatment of blood malignancies [4,5]. Methotrexate, an anti-folate that inhibits dihydrofolate reductase and suppresses the synthesis of thymidine/purine, was prescribed to cancer patients in the 1980s [6,7]. However, despite the initial positive response, patients became resistant to this antimitotic reagent and the disease inevitably relapsed. Since then, extensive drug-screening programs have been performed with the goal to identify new candidate targets for cancer treatment [8]. Furthermore, because of the toxicity associated with the administration of poorly characterized drugs, it became clear that an understanding of the mechanisms through which anti-cancer drugs function is a key element in the pursuit of safe and effective cancer therapies.

### 2.2. Representative Signaling Pathways and Cancer

Since the early discovery of oncogenes (e.g., *MYC*, *RAS*, *BRAF* and *KIT*) and tumor suppressor genes (e.g., *TP53*, *BRCA1* and *PTEN*), cancer-associated genetic lesions have been extensively documented [9,10]. Nowadays, signaling pathways and molecular networks are recognized for their critical roles in executing and controlling important pro-survival and pro-growth cellular processes and are therefore chiefly implicated in the onset of cancer, and also in its potential treatment.

In this review, we will focus on a few signaling pathways and on selected types of cancer for which targeted therapies have significantly contributed to improve clinical outcomes. Two pathways in particular, the PI3K/AKT/mTOR signal transduction pathway and the Ras/MAPK pathway, are frequently activated or mutated in cancer. These cascades are highly interconnected in mediating upstream signals from receptor tyrosine kinases (RTKs) to intracellular effector proteins and cell cycle regulators [11]. In addition to the linear signals propagated from the extracellular space into the cytoplasmic and nuclear compartments, the PI3K and MAPK pathways are strongly interconnected through a number of positive and negative feedback loops. As an example, targeted inhibition of mTORC1 through the administration of analogs of rapamycin, also known as rapalogs, can cause MAPK reactivation and can lead to resistance to single mTORC1 inhibition. Consequently, combinatorial targeting of mTOR plus MAPK has been shown to induce a better response to therapies [12].

Signaling pathways that regulate cell cycle progression (e.g., RTK-CDK4/6) and genomic stability (e.g., BRCA1, p53 and p21) are also frequently altered in cancer. Recent pan-cancer analyses have shown that chromosomal rearrangements, occurring as a result of genomic instability, are early events in various cancer types such as glioblastoma, melanoma, and breast adenocarcinoma [10]. Consistently, CDK4/6 inhibitors and PARP inhibitors have been tested in a number of clinical trials for the treatment of cancer cells with compromised DNA repair, e.g., BRCA1/2 mutations. Furthermore, the growth of cancers expressing hormonal receptors, including breast, ovarian, and prostate cancers, has been shown to largely depend on the proliferative signal induced by their relative hormones, such as estrogen and androgen. The understanding of the mechanisms through which these nuclear hormones and their receptors function has led to the development of the diverse classes of inhibitors currently used as the standard of care for cancer treatment, as we detail below [13,14,15].

Notably, these selected examples do not include successes achieved through therapies directed at additional signaling pathways such as the apoptotic cascades [16]. Moreover, emerging evidence suggests that the β-adrenergic receptor signaling, either at the level of the tumor macro-environment (e.g., adrenal medulla) or within the micro-environment (i.e., innervation by sympathetic nerve fibers within tumor tissues), can promote tumor progression and can be targeted by β-adrenergic antagonists and beta-blockers [17,18]. Thus, these studies collectively show that after decades of intense genetic and biochemical studies, we have been able to identify a number of critical oncogenic alterations and exploit them for cancer treatment. This has tremendously improved our understanding of cancer biology and has also contributed to improving the overall survival rates of a number of cancer patients. Yet despite much progress, too often we find that even the most advanced targeted therapies still fail to effectively put cancer in remission.

## 3. Why Do We Need to Identify Novel Molecular Perturbations?

### 3.1. Targeting Hormone Receptor Signaling through Endocrine Therapies

The first hormone therapy ever applied for cancer treatment was used in 1941 by Dr Charles Huggins, who administered estrogen and removed the testicles to lower the levels of testosterone in patients with prostate cancer [19]. Ever since, endocrine therapies have been largely developed and used for the treatment and management of breast and prostate cancer.

**Figure 1:**
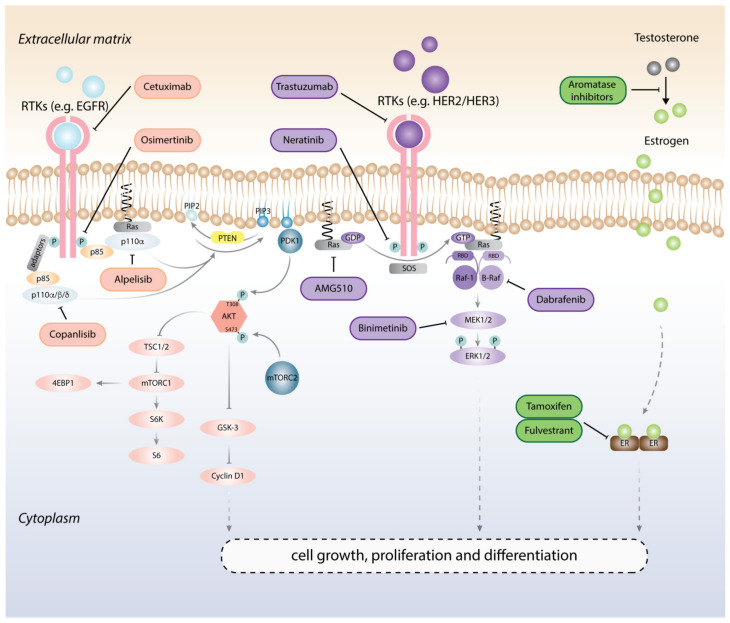
Targeted therapies for cancer treatment. In physiologic conditions, ligands bind to receptor tyrosine kinases (RTKs) at the cell membrane and induce the autophosphorylation of the RTKs’ catalytic domains and the activation of downstream effectors, e.g., p85 release and the activation of the p110 catalytic subunit within PI3K; GDPase–GTPase conversion of Ras. Activation of the PI3K and MAPK pathways initiates a series of phosphorylation events that promote cell growth and proliferation and regulate cellular differentiation. In the presence of estrogen, the estrogen receptor (ER) dimerizes and translocates into the nucleus where it activates pro-growth transcription programs. Examples of FDA-approved monoclonal antibodies and small molecule inhibitors acting on multiples molecular nodes are shown.

Breast cancer (BC) is the second most common type of cancer affecting women worldwide. It is expected that almost 70% of newly diagnosed BC will depend on the pro-growth effect driven by the hormone estrogen and its receptor. The estrogen receptor (ER), which is a nuclear receptor able to transcribe pro-growth genes and to cross-talk with multiple receptor tyrosine kinases (RTK) and signaling pathways to sustain tumor cell growth [20]. Given the high frequency of ER positive (ER^+^) BC [21], therapies suppressing the tumorigenic activity of this hormone are in continuous evolution. Several treatment approaches have been developed to inhibit estrogen action, and these include:

(1) aromatase inhibitors (e.g., letrozole, anastrozole, and exemestane), which inhibit estrogen production and levels [22]
(2) selective estrogen receptor modulators (SERMs) such as tamoxifen and toremifene, which bind to ER and form complexes with co-repressors instead of co-activators, thus halting the ER transcriptional program [14,23]
(3) selective estrogen receptor degraders (SERDs) such as fulvestrant, which bind to ER, prevent its nuclear translocation, and lead to its subsequent degradation (Figure 1) [24,25].

These therapies have dramatically improved outcomes and survival rates of breast cancer patients, and the five-year survival rate for ER^+^ breast cancer is now higher than 90%. However, despite the prolonged positive response to treatments, ER^+^ BC patients also develop resistance to hormone therapies over time, which can occur through multiple mechanisms. Loss of ER expression occurs in 10% of breast cancers, leading to a loss of treatment efficacy. Moreover, even when ER expression is still retained, molecular rewiring of the signaling pathways through the engagement of alternative RTKs such as EGFR [26], HER2 [27], and FGFR [28] can lead to the reactivation of ER-regulated transcription programs and tumor cell growth.

Multiple clinical trials are currently underway to identify combination therapies able to overcome resistance to ER targeted treatments. In an ongoing clinical trial (NCT01953926), the HER2 inhibitor neratinib, administered together with fulvestrant, increased progression-free survival compared to neratinib alone in ER^+^/HER2-mutant metastatic BC patients pre-treated with endocrine therapy [29]. The FGFR inhibitors erdafitinib (NCT03238196) and TAS-120 (NCT04024436) have entered clinical trials in combination with fulvestrant to treat ER^+^ breast cancer with FGF amplification. The PI3K–AKT signaling pathway, which acts downstream of many RTKs, is frequently found constitutively active in BC, owing to mutations in *PIK3CA* and *PTEN* [30]. Consistently, combinatorial use of fulvestrant with the PI3K p110α-specific inhibitor alpelisib has been approved for the treatment of ER^+^ metastatic breast cancer harboring *PIK3CA* mutations [31]. Finally, a recent clinical trial testing the combinatorial effect of fulvestrant plus the AKT inhibitor capivasertib has also started. [32].

Similar to breast cancer, prostate cancer (PCa) can also be driven by high levels of hormones such as androgens, whose synthesis is regulated by the hypothalamus–pituitary–testicular axis [13]. PCa is one of the leading causes of death for men worldwide, and various therapeutic approaches have been developed to monitor and treat this slow-progressing tumor. After an initial active surveillance that can last several years, once the disease progresses from a low-risk and slow-growing tumor to a high-risk aggressive disease, prostatectomy and radiotherapy are generally proposed as the first line treatment [33]. These initial treatments can be further followed up by androgen deprivation therapy (ADT) plus chemotherapy. If prostate cancer progresses to metastatic castration-resistant prostate cancer (mCRPC), this is then treated with antagonists of gonadotropin-releasing hormone and androgen receptor (AR), which, altogether, lower testosterone activities; abiraterone can be included in the treatment to further inhibit androgen synthesis [34,35,36].

Molecular profiling of PCa has identified three main mechanisms of resistance to ADT in CRPC. Hotspot point mutations in the ligand-binding domain of AR, such as the L702H, W742C, H875Y, and T878A mutations, are predominantly found in CRPC samples but not in primary PCa samples. Together with AR amplification, these missense mutations account for 60% of CRPC oncogenic mutations [37] and function by rendering prostate cancer cells resistant to AR antagonists (e.g., hydroxyflutamide and enzalutamide) or by imposing agonist-bound structural conformations, which lead to the reactivation of AR signaling [38,39].

An additional mechanism of resistance to androgen deprivation is associated with residual levels of androgens produced by the activation of the de novo steroidogenesis pathway from cholesterol. Antiandrogen and steroidogenesis inhibitors such as enzalutamide and abiraterone are currently approved agents for CRPC treatment [40,41].

Finally, reports have shown that the activation of other steroid receptors can also contribute to treatment failure and PCa regrowth. The glucocorticoid receptor (GR)-regulated transcriptome highly overlaps with AR gene signatures, and compensatory activation of the GR signaling can lead to enzalutamide resistance in prostate cancer xenograft models [42]. Furthermore, *PIK3CA* mutations and/or loss of PTEN, as well as Raf activation and DNA repair signaling pathways, have all been reported to contribute to the growth of metastatic CRPC through AR-independent mechanisms. Because of these events, a number of combinatorial treatments using ADT plus inhibitors directed at these signaling nodes are currently being tested in several clinical trials [43].

### 3.2. Targeting Receptor Tyrosine Kinases

Under physiologic conditions, RTKs can transduce growth-promoting signals to the cytoplasmic space. In cancer, RTKs can be found amplified, mutated, and constitutively active, thus causing growth signals to be continuously transduced even in the absence of upstream stimuli. To prevent this effect, monoclonal antibodies and targeted inhibitors have been developed.

Monoclonal antibodies (mAb) directed at the ecto-domains of RTKs act by binding and preventing RTKs interactions to their agonists. Cetuximab, a mAb binding EGFR, was the first FDA-approved monoclonal antibody used for the treatment of metastatic colorectal carcinoma [44]. It functions by inducing receptor dimerization and internalization, thus reducing the overall EGFR protein levels on the plasma membrane. Given the frequency of EGFR activation in cancer, additional tyrosine kinase inhibitors, TKIs, directed at the cytoplasmic domain of EGFR have also been developed. To date, three generations of TKIs have been approved for use in clinics, including:

(1) first generation TKIs, gefitinib and erlotinib, which compete with ATP for the kinase domain of EGFR [45,46];
(2) second generation TKIs, e.g., afatinib and dacomitinib, with an improved affinity for the EGFR kinase domain; and
(3) third generation TKIs, such as osimertinib, which covalently bind to cysteine residue on EGFR [47].

The second generation TKIs were shown to prolong the overall survival of patients with NSCLC treated with a first generation TKI, i.e., gefitinib plus chemotherapy [48]. Unfortunately, acquisition of the EGFR T790M missense mutation, occurring in 50% of NSCLC, was found to cause structural changes in the EGFR binding pocket, thus rendering tumor cells resistant to the second generation of TKIs and requiring new treatments [49]. The EGFR T790M-specific inhibitor osimertinib irreversibly binds to the C797 residue in the ATP-binding pocket of EGFR and has been found to improve progression-free survival (PFS) in patients with the acquired T790M mutation [50,51]. Resistance to EGFR-targeted therapies, including osimertinib, can however still arise due to the activation of parallel RTKs such as HER2 [52], MET, IGFR, FGFR, and PDGFR [53,54], or through the activation of downstream signaling pathways such as the PI3K–AKT and Ras–ERK pathways [55,56]. Combinatorial targeting of EGFR and components of these signaling pathways may be a potential way of overcoming drug resistance to EGFR inhibition.

Human epidermal growth factor receptor 2 (HER2) is an EGFR family member frequently overexpressed in breast and gastric cancer [57]. HER2 is an orphan receptor that does not have a direct ligand. When overexpressed, HER2 can form homodimers or heterodimers with other members of the EGFR family, and transduces the oncogenic signal to the PI3K–AKT and the Ras–ERK pathways. To target HER2 dimerization, and prevent its activation, mAbs such as trastuzumab and pertuzumab have been developed [58,59]. HER2 can also be targeted with trastuzumab emtansine, which is a conjugate between trastuzumab and the cytotoxic reagent emtansine used to treat HER2^+^ breast and lung cancers [60,61]. Bispecific antibodies such as ZW25, which binds to two HER2 epitopes (i.e., trastuzumab-binding domain and pertuzumab-binding domain) [62], and MCLA-128, which targets HER2 and HER3 [63,64], are now in clinical trials. These bispecific antibodies enhance HER2 internalization and better abrogate the HER2 oncogenic signal compared to trastuzumab [65]. Finally, a more recent bispecific antibody, GBR1302, has been found to bind HER2 and CD3, and to direct cytotoxic T cells to HER2^+^ breast cancer cells [66].

In addition to mAbs, reversible TKIs, such as lapatinib, and the irreversible neratinib (pan-HER inhibitor) are also currently used in the clinic to halt signal transduction by blocking the phosphorylation of the HER2 kinase domain [67,68]. However, due to the redundancy in signal transduction mediated by the many members of the HER family, incomplete inhibition of the signal can lead to resistance to targeted therapies. To overcome this, a combination of dual TKI lapatinib, or pan-TKI neratinib, plus mAbs has been shown to comprehensively block the known compensatory HER2 signaling network [69,70]. Recently, tucatinib, a highly selective HER2 TKI, has been shown to inhibit HER2/HER3-mediated MAPK and PI3K/AKT signaling [71]. It suppresses tumor growth and leads to tumor regression in HER2^+^ BT-474 breast xenograft models when used as monotherapy, or together with trastuzumab. Tucatinib has been recently approved by the FDA in combination with trastuzumab and capecitabine, an anti-metabolite, for the treatment of metastatic HER2^+^ breast cancer [72].

Activating HER2 point mutations (e.g., L755S, T862A, and T798M), and the truncated p95HER2 mutation, has been associated with resistance to HER2 therapies in breast cancer patients [73,74,75]. Trastuzumab deruxtecan, an antibody-topoisomerase I inhibitor conjugate, effectively inhibits AKT phosphorylation, induces DNA damage, and suppresses the growth of HER2^+^ patient-derived xenografts (PDX) resistant to trastuzumab emtansine. Trastuzumab deruxtecan is now approved for the treatment of HER2^+^ breast cancer and gastric cancer patients [76,77].

Finally, alterations in PI3K and MAPK pathways have been shown to cause resistance to HER2-based therapies [78,79], and phase III clinical trials are now underway to treat advanced HER2^+^ breast cancer harboring *PIK3CA* mutations using the PI3K p110α-specific inhibitor alpelisib (NCT04208178).

### 3.3. Targeted Therapies Directed at the PI3K/AKT Signaling Pathway

The phosphoinositide 3-kinase (PI3K) signaling pathway plays an essential role in the regulation of cell proliferation, cell growth, and survival downstream of many RTKs (e.g., EGFR family; the insulin receptor, IR; and the insulin-like growth factor receptor, IGFR). RTKs can bind directly to PI3K or can signal through adaptor proteins such as the insulin receptor substrate 1 (IRS1). Multiple classes of PI3Ks exist but class IA PI3K is the most frequently mutated in cancer [80]. Class IA PI3K (from now on just PI3K, unless specified) is a heterodimer consisting of a catalytic subunit, p110; and a regulatory subunit, p85. Upon binding to phospho-tyrosine residues on RTKs or IRS, p85 releases its inhibitory effect and activates p110, which in turn binds and phosphorylates the phosphatidylinositol-4,5-biphosphate (PtdIns-4,5-P2) to generate the second messenger phosphatidylinositol-3,4,5-triphosphate (PtdIns-3,4,5-P3 or PIP3) [81,82]. At the membrane, PIP3 recruits multiple downstream targets such as PDK1 and mTORC2, which catalyze AKT phosphorylation on Thr308 and Ser473, respectively [83,84,85,86]. AKT is a central pro-survival signaling hub that regulates a plethora of downstream effector proteins including the mTORC1 complex, the FOXO family of transcription factors, and cyclin D1, and supports cell growth and proliferation. Multiple tumor suppressor proteins have been shown to limit PI3K pathway activation, such as the lipid and protein phosphatase PTEN [80], the promyelocytic leukemia protein PML [87], and the tuberous sclerosis complex TSC [88].

#### 3.3.1. Targeting PI3K

Many members of the PI3K pathway are mutated in cancer, but *PIK3CA* and *PTEN* are the two critical players with the highest frequencies of alterations. *PIK3CA*, the gene encoding the p110α catalytic subunit of PI3K, is frequently mutated or amplified in endometrial, breast, ovarian, and colorectal cancer [89]. Hotspot mutations in *PIK3CA* have been mapped to the exon 20, encoding the kinase domain (e.g., *PIK3CA* H1047R mutation) and the exon 9, encoding the helical domain (e.g., *PIK3CA* E542K, E545K mutations) [90]. These missense mutations render PI3K constitutively active and are frequently correlated with AKT activation [91,92].

Alterations in the tumor suppressor *PTEN* [93] are found in somatic cancers such as breast, endometrial, prostate, and brain cancer [57], but they can also occur in the germline. where they cause various cancer-predisposition syndromes known as PTEN hamartoma tumor syndromes (PHTS) [94]. In addition to genomic loss or silencing, loss of PTEN function can frequently occur through acquisition of missense mutations [95]. Importantly, through the generation of PTEN knock-in (KI) mouse models, our group and others have demonstrated that two frequent cancer-associated and loss-of-function *PTEN* mutations (i.e., PTEN G129E and C124S) can dimerize with wild-type PTEN and inhibit its function in a dominant negative manner [96,97,98]. In vivo, these *PTEN* mutations cause more rapid tumor formation and progression than that observed in mice with *PTEN* heterozygous condition (*PTEN^+/−^* mice) [96,99], suggesting that patients harboring these *PTEN* alterations may display heightened sensitivity to therapies directed at PI3K and/or AKT inhibition.

Early preclinical studies with the PI3K inhibitors Wortmannin [100,101] and LY294002 [102] suggested that the complete inhibition of all PI3K isoforms could provide a positive response to treatments in *PIK3CA*-mutant cancers. Over the years a number of pan-PI3K inhibitors such as buparlisib [103], pictilisib [104], pilaralisib [105], and copanlisib [106] have been generated and also entered clinical trials. However, due to off-target toxicities, many of them have been discontinued, except for copanlisib, which has been approved by the FDA for the treatment of B-cell lymphomas with an altered PI3K pathway [107]. Copanlisib is also in a phase II trial for the treatment of ER^+^/HER2^-^ metastatic breast cancer patients (NCT03803761).

In order to reduce toxicity and increase treatment efficacy, isoform-selective PI3K inhibitors have been generated. The p110δ-specific inhibitor idelalisib, the first FDA-approved PI3K-isoform specific inhibitor, is nowadays used to treat chronic lymphocytic leukemia [108]. More recently, the PI3Kα-specific inhibitor alpelisib has been approved for the treatment of ER^+^/HER2^−^ *PIK3CA*-mutant and metastatic breast cancer in combination with fulvestrant [31]. Taselisib is another PI3K inhibitor that equally inhibits p110α/γ/δ and has a 10-fold lower activity towards p110β [109]. Preliminary results have suggested that taselisib suppresses the growth of *PIK3CA*-driven xenograft tumors and decreases both the activity and the levels of mutant p110α in vitro [110]. Taselisib is now in a phase II clinical trial (NCT04439175) for both lymphomas and solid cancers with *PIK3CA* mutations.

The PI3K signaling pathway is frequently activated in solid tumors that have become insensitive to endocrine and RTK-targeted therapies. Because almost 40% of ER^+^ breast cancers harbor *PIK3CA* mutations [30,111], combination therapies using alpelisib or taselisib with endocrine therapies have been extensively tested in clinical trials [112,113]. However, targeted PI3K inhibition has been shown to induce systemic metabolic adaptation, which impacts drug efficacy and limits tumor response to treatment [114]. Because of this, PI3K-mutant specific inhibitors are now under development. GDC-0077, a PI3Kα inhibitor more selective towards mutant PI3K than wild-type PI3K, was found to induce proteasomal degradation of mutant PI3K, and caused tumor regression in *PIK3CA*-mutant breast cancer xenografts [115,116]. This compound is now in clinical trial (NCT03006172) as a single agent, as well as in combination with other TKIs and endocrine therapy for the treatment of PIK3CA-mutant solid tumors [117].

Multiple mechanisms of resistance have been reported to cause treatment failure of PI3K inhibitors. For instance, the rewiring of the downstream signaling cascade can involve the reactivation of molecular targets such as mTOR [118], Pim1 [119], PDK1/SGK1 [120], or SGK3 [121,122], all reported to support growth in an AKT-independent manner. Reactivation of additional p110 isoforms such as p110β has also been shown to sustain PIP3 accumulation and attenuate the growth inhibitory effect induced by p110α inhibition in *PIK3CA*-mutant and *HER2*-amplified breast cancer cells [123]. Consistently, pre-clinical studies have shown that combined p110α and p110β inhibition can better suppress growth and induce tumor regression in PTEN-deficient breast and prostate cancer models than alpelisib alone [124,125].

*PIK3CA* mutations can co-occur with activating mutations in RTKs such as EGFR, KRAS, and ALK in lung adenocarcinomas [126], as well as with the loss of PTEN in endometrial and breast cancer [127,128,129]. Juric et al. reported that *PIK3CA*-mutant breast cancer patients treated with alpelisib became refractory to treatment upon the acquired loss of PTEN [130]. Treatments with the p110β inhibitor AZD6482 and alpelisib were shown to resensitize tumor cells and suppress the growth of PTEN-mutant PDX xenograft models [130]. However, a recent clinical trial testing the efficacy of alpelisib with aromatase inhibitors (letrozole or exemestane) for PI3K-mutant ER^+^ metastatic breast cancer demonstrated that *PTEN* deletion, or loss-of-function *PTEN* mutations (e.g., *PTEN* A126S and PTEN R130 *) together with *ESR1* activating mutations can still cooperate to cause treatment resistance [131].

Due to the high frequency of heterozygous deletion found in the *PTEN* genomic locus, reactivation of the remaining PTEN wild-type proteins has been considered an attractive approach for cancer therapy. The Pandolfi group has recently found that the ubiquitin E3 ligase 1 (WWP1) mediates PTEN poly-ubiquitination on lysine 27, which prevents PTEN dimerization, plasma membrane translocation, and activity toward PIP3. In turn, this abolishes PTEN tumor suppressive activity and favors cancer progression in a Myc-driven prostate cancer model [132]. Authors further showed that indo-3-carbinol, a natural WWP1 inhibitor, can inhibit AKT activation and induces apoptosis in prostate organoids and tissues derived from Hi-Myc mutant mice, suggesting that WWP1 inhibition through the restoration of PTEN activity is synthetically lethal in Myc-driven tumors.

#### 3.3.2. Targeting AKT

The proto-oncogene AKT, also known as protein kinase B, encompasses three isoforms (i.e., AKT1, AKT2 and AKT3), and promotes mTOR activation while suppressing p21/p27-mediated apoptosis [133]. Constitutive activation of AKT, due to activating mutations in upstream RTKs, PI3K, and/or loss-of-function PTEN mutations, has been implicated in multiple cancers [90,96,134,135]. Increased AKT activation has also been associated with resistance to chemotherapy [136] and other TKIs [137], making AKT an attractive target for combination treatments. Ipatasertib (GDC-0068), an ATP-competitive pan-AKT inhibitor, synergizes with chemotherapeutic agents to inhibit growth and induce apoptosis in xenograft models of a spectrum of cancer cell lines, including breast, prostate, ovarian, and NSCLC cells [138]. Moreover, *PTEN* deletion has been shown to sensitize MCF-10A breast cells and tumor xenografts to ipatasertib, suggesting that the loss of PTEN function can be a biomarker predicting the response to AKT inhibitors [138]. Consistent with this, we have recently demonstrated that loss-of-function *PTEN* mutations (*PTEN* C124S/+ and *PTEN* G129E/+) coupled with oncogenic PI3K (*PIK3CA* H1047R) lead to AKT hyper-activation in a mouse model of breast cancer and that, although resistant to alpelisib, PTEN and PI3K mammary organoids are sensitive to the AKT inhibitor MK-2206 [139].

Based on this large set of evidence, multiple clinical trials are now underway to evaluate the efficacy of combination therapies using AKT inhibitors with additional targeted therapies [140,141].

### 3.4. Targeted Therapies Directed at the Ras/MAPK Pathway

Activation of RTKs, such as the EGFR family, promotes activation of the guanosinetriphosphatase (GTPase) Ras proteins, which switch from their inactive Ras–GDP loaded state to the active Ras–GTP state. The Ras guanine nucleotide exchange factors (GEFs) catalyze the loading of GTP to RAS, whereas GTPase-activating proteins (GAPs) hydrolyze GTP–Ras to the GDP-bound inactive state [142,143]. Ras activation recruits and activates downstream targets such as Raf, including A-Raf, B-Raf, and Raf-1, which are recruited to the plasma membrane through their Ras-binding domain (RBD) for dimerization and activation. Active Raf phosphorylates and activates MEK and ERK kinases [144,145,146], leading to ERK1/2 nuclear translocation and the promotion of transcriptional programs supporting cell growth, proliferation, or differentiation. 

Multiple members of the MAPK pathway are mutated in cancer. *KRAS* is frequently mutated in solid tumors such as pancreatic ductal adenocarcinomas (82%), colorectal carcinomas (41%), and lung adenocarcinomas (32%) [147]. *BRAF* is mutated in 8% of human cancers, primarily melanoma, whereas *MEK* and *ERK* are rarely mutated. B-Raf/Raf-1 heterodimer is the predominant form of heterodimers transducing the Ras signal in cancer [148,149]. The most prevalent activating mutation in BRAF occurs at the V600 position, which imposes an active conformation and can also transduce pro-growth signals as monomers in a Ras-independent manner.

The GTPase K-Ras oncoproteins have long been considered undruggable targets; however, recent developments have shown that new selective compounds can form covalent bonds with mutant KRAS^G12C^, which accounts for 13% of all *KRAS* mutations, and inhibit KRAS activity. AMG510 and MRTX849 are two KRAS^G12C^ inhibitors currently being tested in clinical trials [150,151].

Three B-Raf inhibitors (i.e., dabrafenib, encorafenib, and vemurafenib) are available for the treatment of melanoma and NSCLC harboring the BRAF^V600E/K^ mutations [152]. B-Raf inhibitors specifically inhibit monomeric BRAF^V600^ mutant while their inhibitory potential towards dimeric Raf is reduced. Unexpectedly, B-Raf inhibitors have been reported to activate, instead of inhibit, the MAPK signaling in non-BRAF^V600E^ tumor cells. It was found that by binding to B-Raf, these inhibitors increased the potential of wild-type Raf-1 to heterodimerize with B-Raf [153], thus sustaining high levels of pERK1/2 in a Ras-dependent manner [154,155]. 

In order to block the MAPK signaling in non-BRAF^V600^ tumor cells, type-II RAF inhibitors have been developed. Type-II RAF inhibitors can bind to B-Raf dimeric kinases and prevent Raf-1 transactivation, thus blocking downstream activation of MEK and ERK [156,157,158]. Type-II Raf inhibitors such as PLX704 and PLX8394 are also called ‘paradox breaker’. They disrupt the formation of B-Raf homodimers/heterodimers and block the B-Raf-driven ERK signaling cascade in both BRAF^V600^ mutant and non-BRAF^V600^ mutant tumor cells [159,160].

Type-II Raf inhibitors can also overcome the ERK reactivation caused by vemurafenib treatments in vemurafenib-resistant melanoma cell lines [159]. A pre-clinical study has shown that in combination with cetuximab, PLX8394 completely inhibited the growth of colorectal cancer PDXs with BRAF K601E and G466V mutants and resistant to vemurafenib [160].

Although the first generation of type-II RAF inhibitors (e.g., sorafenib and LY3009120) did not show significant activities in clinical trials, a new generation of pan-RAF inhibitors, e.g., AZ628, TAK632 and LXH254, have been developed and have been demonstrated to reduce phospho-MEK and phospho-ERK levels in the non-BRAF^V600^ mutant lung cancer H1666 cell line [161], in BRAF mutant human melanoma xenograft models [162,163], and human NSCLC xenograft models [164].

Resistance mechanisms to Raf inhibitors are usually due to genetic alterations within the MAPK pathway, such as *KRAS/NRAS* mutations, *BRAF^V600E/K^* amplification, and *MEK1/2* amplification [165,166]. Since Ras–GTPase activates both the MAPK and PI3K signaling pathways, activating mutations in the PI3K signaling pathways such as *PTEN* deletion and the constitutive activation of PI3K and AKT, it has been associated with resistance to Raf inhibition [167]. Because RAS mutations can co-exist with mutations in members of the PI3K pathway [168], a simultaneous inhibition of both the MAPK and PI3K signaling pathways may overcome resistance to Raf inhibitors. Pre-clinical studies have shown that pan-PI3K inhibitors, AKT inhibitors, or mTOR inhibitors, in combination with MEK inhibitors, may deliver promising tumor suppressive effects in KRAS or BRAF-mutant tumors; however, the associated toxicity may still limit clinical benefits [169,170,171,172]. An alternative approach to target both the PI3K/AKT and MAPK pathways is to combine the IGFR inhibitor, linsitinib, with the KRAS^G12C^ specific inhibitor, ARS-1620, and the mTOR inhibitor, everolimus [173]. This three-drug combination was found to be better tolerated in clinical settings when each drug was administered at low dose [173].

Currently, there are three MEK1/2 inhibitors (binimetinib, trametinib, and cobimetinib) available for the treatment of melanoma and NSCLC harboring the *BRAF^V600E/K^* mutations [152]. Next-generation MEK inhibitors are also available, such as RO5126766, which is defined as a dual Raf/MEK inhibitor that complexes with both proteins and inhibits phosphorylation of ERK in NRAS melanoma cells and in KRAS mutant xenograft models [174,175]. This compound is currently under clinical trials to treat RAS mutant NSCLC (NCT03681483), and colorectal and ovarian cancer (NCT03875820). Since resistance to MEK inhibitions is usually due to reactivation of downstream ERK1/2 [167,176], multiple ERK inhibitors such as ulixertinib (NCT04566393) and LY3214996 (NCT04391595) are also in clinical trials and may be considered as a valuable option to overcome resistance to FDA-approved MEK inhibitors [177].

### 3.5. Targeted Therapies Directed at Cyclin-Dependent Kinases

**Figure 2:**
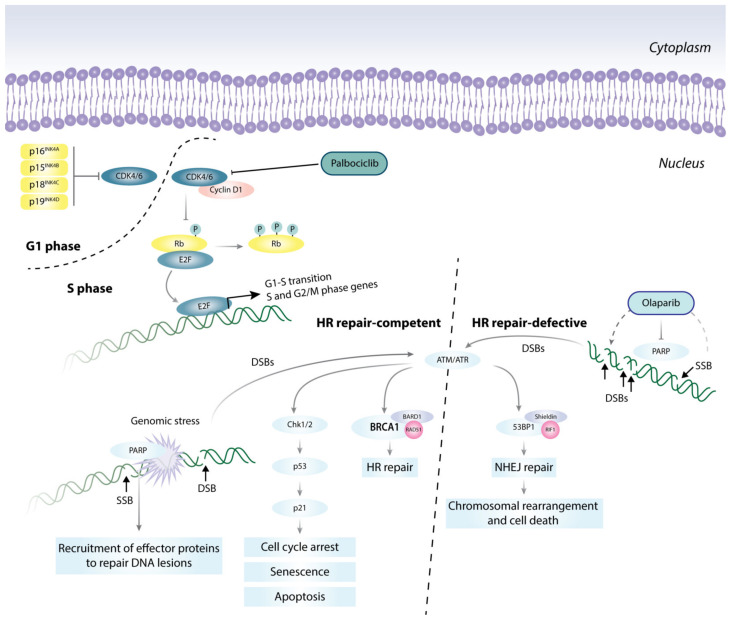
Targeted therapies directed at cell cycle and DNA damage repair pathways. Pro-growth stimuli promote the formation of the CDK4/6-cyclin D1 complex, which phosphorylates and inactivates the tumor suppressor Rb, and releases E2F. E2F then initiates a transcription program that supports G1-S phase transition. Genomic stress activates DNA damage response (DDR) mechanisms, e.g., ATM/ATR, and PARP signaling pathways, and triggers the Chk1/2-p53-p21 axis to induce cell cycle arrest, senescence, or apoptosis as a means to halt the propagation of genetic lesions. Accumulation of DNA double-stranded breaks (DSB) can be repaired by two mechanisms: (1) the error-free homologous recombination, HR; or (2) the error-prone non-homologous end-joining, NHEJ. FDA-approved PARP inhibitors, e.g., Olaparib, when used with standard treatments, can force HR repair-defective cells, such as in BRCA1 mutant cells, to activate the error-prone NHEJ pathway, leading to genomic instability beyond repair and causing cell death. BARD1 (BRCA1-associated RING domain protein 1); RIF1 (Rap1-interacting factor 1 homolog); and 53BP1 (TP53-binding protein 1).

Cyclin-dependent kinases (CDKs) are kinases that complex with cyclins to initiate and regulate cell cycle progression. CDK activation is cell-cycle phase specific and is regulated by a few inhibitory proteins such as p16^INK4A^ (*CDKN2A*), p15^INK4B^ (*CDKN2B*), p18^INK4C^ (*CDKN2C*), and p19^INK4D^ (*CDKN2D*) [178]. Upon growth factor stimulation, the CDK4/6 complex binds to cyclin D1 and phosphorylates the retinoblastoma protein (RB), a master regulator of cell cycle entry and critical tumor suppressor (Figure 2). RB phosphorylation inhibits RB function and releases its inhibitory effect on the E2F transcription factor, thus promoting G1-S cell cycle transition [179,180]. The CDK4/6 complex also activates FOXM1-mediated transcription, which plays an important role in the regulation of cell cycle progression and the suppression of cellular senescence [181].

Inhibitors targeting CDK4/6 activation (CDK4/6i) have made important progresses in breast cancer treatment. To date, there are three FDA-approved CDK4/6i in clinics: abemaciclib, ribociclib [182,183,184], and palbociclib [185]. Except for abemaciclib, which can be used as monotherapy in refractory ER^+^/Her2^-^ breast cancers [186], ribociclib and palbociclib are currently used in combination with aromatase inhibitors, or with fulvestrant for ER^+^ luminal breast cancers [182,187]. Emerging evidence has shown that CDK4/6i can also regulate the host immune response and could therefore be used in combination with immune checkpoint inhibitors [188,189].

CDK4/6 inhibition effectively reverses drug resistance to endocrine therapies and RTK-based therapies. A screening of 47 breast cancer cell lines revealed that CDK4/6 inhibition suppresses the growth of ER^+^ luminal cancer and HER2^+^ cancer cell lines by inhibiting RB phosphorylation. More importantly, in combination with tamoxifen, palbociclib also resensitizes resistant MCF7 cells to tamoxifen [190]. Miller and colleagues have shown that chronic deprivation of estrogen can lead to the hyper-activation of the PI3K/AKT signaling pathway and sustained CDK4-E2F activation, thus causing estrogen resistance in breast cancer cells [191]. Consistently, a combinatorial drug screen of 42 compounds found that CDK4/6 inhibition exhibits the most significant synergism with PI3K inhibitors (BYL719 and GDC-0941) in a panel on PI3K-mutant breast cancer cell lines (T47D, MCF7, and MDA-MB-453) resistant to PI3K inhibition [192]. Molecular studies found that high levels of pRB S780 may contribute to the resistance to PI3K inhibition in resistant cells in vitro, and in derived xenograft models. In agreement with this, combinatorial use of the CDK4/6 inhibitor LEE011 plus BYL719 or GDC-0941 caused tumor regression in breast cancer xenograft models [192].

Goel et al. have shown that CDK4/6 inhibition can also resensitize HER2-resistant breast cancer cells to trastuzumab, and that a combination of CDK4/6i abemaciclib with trastuzumab induces tumor regression in a HER2-driven breast cancer mouse model [193]. Similarly, CDK4/6i can resensitize therapy-resistant tumors to EGFR inhibitors, MET/TRK inhibitors, and MEK inhibitors by suppressing the growth of tumor cells in multiple pre-clinical models, in vitro and in vivo [194,195,196,197].

Rewiring of cell-cycle regulators or activation of alternative pathways can however cause resistance to CDK4/6i [198]. Although the status of *RB* can be used to predict sensitivity to CDK4/6i, genetic alterations in *TP53* [199], *AKT1* and *RAS* [200], as well as overexpression of *CDK4/6* [199,201], *CNNE1/2* [200,202], and *CDKN2A/2B* [203], have been associated with CDK4/6i resistance. Amplifying or activating mutations in *FGFR1/2* and *ERBB2* [28,200] have also been found to cause resistance to CDK4/6i. Genetic alterations in the Ras–ERK pathway and Hippo pathways may contribute to CDK4/6i resistance in prostate [204] and breast cancer [199], respectively.

A recent study further revealed that loss of PTEN expression is a mechanism of adaptive resistance to CDK4/6i in breast cancer patients [205]. Authors found that the PTEN knock-out (KO) in T47D cells caused resistance to ribociclib and palbociclib, but that administration of the AKT inhibitor MK-2206 resensitized PTEN-KO T47D cells to CDK4/6i, suggesting that AKT activation mediates the resistance to CDK4/6i in PTEN-deficient cells. Consistent with this, combinatorial treatment of ribociclib with MK-2206 induced tumor regression in PTEN-null T47D cells xenograft models [205].

### 3.6. Targeting Genomic Instability

DNA replication is a dynamic process under the active surveillance of cell cycle checkpoint inhibitors, which maintain the integrity of the genetic information during cell division. When DNA damage occurs in normal cells, multiple DNA damage response (DDR) pathways and mechanisms are induced. For instance, the base-excision repair (BER) and the nucleotide-excision repair machineries mediate single base-pair repair [206]; homologous recombination (HR) and non-homologous end joining (NHEJ) can mediate repair on DNA double-strand breaks (DSB) [207,208,209]; and mitotic checkpoints ensure the correct centrosome localization and chromosomal segregation during cell division [210]. Defects in these DNA repair mechanisms can lead to the induction of failsafe mechanisms, which act as additional checkpoints by inducing either a senescence response, or cell death [211].

However, upon the deletion of tumor suppressor genes or the activation of oncogenes, genomic instability can accumulate within cells and malignant transformation occurs. Mutations in DNA repair genes (e.g., *BRCA1/2*, *PALB2*, and *RAD51C*) and genome gatekeeper genes (e.g., *TP53*, *ATM* and *CHEK2*) are frequently found in cancer [212,213], and cancer cells with defective DDR or cell cycle checkpoints replicate damaged DNA to meet the uncontrolled proliferative drive induced by oncogenic signals.

Nevertheless, we have learnt how to exploit DNA damage accumulation by targeting DDR pathways and inducing mitotic catastrophe of cancer cells. Topoisomerase inhibitors (e.g., topotecan and etoposide) and DNA alkylating agents (e.g., cyclophosphamide and cisplatin) have long been used to induce cancer cell death [214], but targeted therapies directed at DDR pathways are still limited in use.

#### 3.6.1. Targeting PARP to Exploit Defective HR Repair in Cancer

DNA double-strand breaks (DSB) can be repaired through either the error-free HR [215,216], or through the error-prone NHEJ repair mechanisms [217]. In BRCA-expressing cells, HR is the predominant mode of DNA damage repair in the S/G2 phase. During DNA replication, DSBs are repaired by RAD51 recombinase, which uses the homologous sequence of the sister chromatid as template DNA. This process is facilitated by the BRCA1/BARD1 (BRCA1-associated RING domain protein 1) and BRCA2/PALB2 complexes [218]. Unlike HR repair, the NHEJ machinery depends on the function of proteins such as Ku70/80 and the DNA-dependent protein kinase catalytic subunit (DNA-PKc) complex, which recruit ligases to repair DNA breaks [217]. NHEJ is generally prone to introducing DNA deletions or insertions and is not used as the preferred pathway when a wild-type BRCA gene is expressed to ensure DNA integrity [219]. However, when HR repair is not functional, such as upon BRCA loss, poly(adenosine diphosphate-ribose)ylation (PARylation) and NHEJ repair become the main DNA repair mechanisms [220]. Defective HR repair is associated with germline mutations in *BRCA1* and *BRCA2*, which frequently occur in hereditary breast and ovarian cancer [221]. These mutations increase the lifetime risk of developing breast cancer from 12% to 46–87%, and ovarian cancer from 2% to 39–63%. *BRCA2* pathogenic mutations are also associated with a 3.7-fold increase in the risk of developing prostate cancer.

PARylation is an early signal for sensing DNA damage. When cells undergo base-excision repair, the PAR polymerases, PARP1 and PARP2, are mobilized to DNA single-strand breaks where they synthesize branched PAR on acceptor proteins to stabilize the replication fork and facilitate the recruitment of DNA repair factors [222,223]. PARP inhibitors (PARPi), now in multiple clinical trials, act by blocking PARP1 polymerase activity, which in turn traps and blocks the replisome from proceeding at the site of the DNA damage [224]. This condition increases the load of the DNA damage in BRCA-mutated HR-deficient tumor cells, and leads to chromosomal abnormalities and mitotic catastrophe [220]. PARPi such as olaparib, niraparib, talazoparib, and rucaparib have been approved for the treatment of HR-deficient breast and ovarian cancers [225], and since May 2020, they can also be used for the treatment of metastatic, castration-resistant prostate cancer [226].

However, a large fraction of human cancers are HR competent and are therefore unresponsive to PARP inhibition. To sensitize HR-proficient tumor cells to PARP inhibitors, a new combination treatment with the PI3K inhibitor has been tested. The pan-PI3K inhibitor BKM120 has been shown to induce the ETS-mediated downregulation of BRCA1/2, which depletes nucleotide triphosphate synthesis [227,228]. Reduced nucleotides synthesis induces DNA damage and synergizes with PARP inhibitors in both HR-proficient and -deficient breast cancer mouse models. Based on pre-clinical results, a recent phase 1b trial has demonstrated synergism between olaparib and the PI3K p110α-specific inhibitor alpelisib in treating HR-proficient epithelial ovarian cancers [229]. A recent study has also shown that simultaneous inhibition on PARP and EZH2, which mediates epigenetic silencing and suppresses NHEJ activity, can inhibit the growth of HR-proficient ovarian PDX models by inducing the NHEJ pathway and chromosomal abnormalities beyond repair, leading to apoptosis [230].

Resistance to PARPi is usually caused by the reactivation of the HR repair pathway. For example, restoration of BRCA1/BRCA2 activity due to reversion mutations, promoter demethylation [231,232], or the amplification of the mutant *BRCA2* allele [233] have all been associated with cancer resistance to PARPi. Additional proposed mechanisms of resistance to PARP inhibition in HR-deficient tumor cells have been recently reviewed [234].

In light of the resistance to PARP inhibitors, researchers have tried to combine PARPi with inhibitors that target the ATM (ataxia-telangiectasia mutated) and ATR (ATM- and Rad3-related) pathways, in order to block DDR pathways on multiple levels [235,236]. Pre-clinical studies and clinical trials using replication/mitotic checkpoint inhibitors such as CHK1 inhibitors (UCN-01, LY2606368) [237], ATR inhibitors (AZD6738), AURKA inhibitors (ENMD-2076), PLK inhibitors (Volasertib), and TTK inhibitors (BAY1161909) [238] are currently being tested in combination with other inhibitors with the goal of causing replication stress and cell death of cancer cells [239,240,241].

Finally, PARylation turnover is a process regulated by poly(ADP-ribose) glycohydrolase (PARG), which degrades PAR chains to promote the firing of replication fork and cell cycle progression once DNA repair is complete [242,243]. Inhibiting PARG would lead to the over-accumulation of PAR chains and eventually cause replication fork collapse and cell death [244,245]. Pillay et al. [246] demonstrated that the PARG inhibitor PDD00017273 [247] showed synthetic lethality with the inhibition of a number of G2/M checkpoint proteins such as TIMELESS, HUS1 RFC2, ATR, WEE1, and CHK1. These combination treatments can cause replication catastrophe independent of BRCA1/2 status and therefore could be considered as an option to kill HR-proficient cells.

#### 3.6.2. Targeting p53 and p21 to Induce Synthetic Lethality

TP53 is the most frequently mutated tumor suppressor gene in human malignancies, and because of its essential role in regulating genome stability it is also known as the guardian of the genome [57,248]. Under physiologic conditions, p53 protein levels are restricted by the E3 ligase MDM2. However, upon cellular stress, the binding between MDM2 and p53 is disrupted, and p53 accumulation and activation occurs. The tumor suppressor p53 is a transcription factor that regulates the expression of critical genes such as *CDKN1A*, also known as p21, which induces cell cycle arrest and senescence, and BAX and PUMA, which promote apoptosis. Because of its central role in regulating cell cycle progression, forced p53 expression has long been considered a potential therapeutic approach for cancer treatment. Small molecule inhibitors such as nutlins and di-hydroisoquinolinones can disrupt the binding of MDM2 to p53, and their use has induced positive response in tumors retaining wild-type p53. Some of these compounds have entered clinical trials [249].

An alternative approach to exploiting p53 expression levels for cancer treatment is to identify contexts in which synthetic lethality can be achieved. For instance, ATR has been shown to maintain genomic stability by delaying cell cycle progression and preventing replication forks from collapsing [250]. However, when ATR is inhibited, its downstream effector p53 becomes the predominant DDR mediator. Consistently, the ATR inhibitor AZD6738 was found to cause DNA damage followed by mitotic catastrophe in a p53- or ATM-defective context, as observed with chronic lymphocytic leukemia (CLL) cells [251]. Moreover, p53 can promote 53BP1 (TP53-binding protein 1) recruitment to sites of DNA damage, thus excluding BRCA1 and inducing NHEJ [252]. In p53-defective cells, reduced 53BP1 recruitment is accompanied by an increase of BRCA1 recruitment to sites of DNA damage, thus making HR the preferable mechanism of DNA repair. This suggests that p53-mutant cells may be sensitive to HR inhibition [253].

The CDK inhibitor p21^WAF1/CIP1^ is one of the p53-regulated genes mediating cell cycle arrest, apoptosis, and senescence in response to DNA damage [254]. Histone deacetylase inhibitors can increase p21 expression and, when used in combination with cytotoxic agents, can induce cell death or cell cycle arrest in cancer cells such as hepatocellular carcinoma, ovarian cancer, and prostate cancer [255,256]. Synthetic lethality has also been used to target p21. For example, p53-null colorectal cancer cells with low p21 expression are more sensitive to CHK1 inhibition [257].

However, new and conflicting data have shown that high p21 levels may also occur in cancer, which correlates with poor disease outcomes [258,259,260]. A recent study has discovered a new population of Ki67^+^ dividing cells expressing high levels of p21 in head and neck squamous cell carcinomas, lung squamous cell carcinoma, and urothelial carcinomas [261]. In addition, using the p53-null Saos-2 osteosarcoma cell line and p53-deficient Li–Fraumeni cell lines, Galanos et al. showed that high p21 levels influence the function of the CRL4–CDT2 ubiquitin ligase and increase levels of the licensing factors CDC6 and CDT1, which lead to defective origin licensing and replication stress, fueling genomic instability in cancer cells. It was found that cells with prolonged high p21 levels would eventually escape senescence and display aggressive phenotypes such as increased invasion and genotoxic drug tolerance. Mechanistically, high levels of p21 favored Rad52 recombinase-dependent break-induced replication (BIR) and single-strand annealing (SSA) as the main DNA repair mechanism over error-free synthesis-dependent strand annealing (SDSA) [262]. This suggests that targeting Rad52 may be synthetically lethal with high p21 levels in p53-null cancers. Additionally, clinically used treatments such as dexamethasone have also been found to induce p21 expression independent of p53, suggesting a detrimental use of this compound in patients with p53 deficiency. Indeed, both p53 mutations and high p21 levels have been demonstrated to exert oncogenic activities in cancer in a context dependent fashion [263,264].

### 3.7. Two Is Better Than One

Here we have summarized a number of known targeted therapies that affect the function of essential molecular hubs and are used to deliver better anti-cancer treatments. However, as we have detailed with various examples, signaling adaptation, acquired resistance, and novel defense mechanisms still allow cancer cells to survive and relapse. We propose that a better understanding of the “escape” mechanisms developing in cancer cells under treatment can provide new insights that can inform more effective combination therapies and will hopefully put cancer in a more stable remission.

## 4. How Can Technological Advances Assist with the Identification of New Cancer Targets and the Delivery of Cancer Drugs?

### 4.1. Phosphoproteomics and Kinome Profiling

While genomic and transcriptomic sequencing projects have allowed the identification of genetic mutations and gene expression profiles associated with tumor initiation and progression, the molecular changes affecting the activation status of signaling pathways during cancer evolution still remain to be characterized. To identify pathological alterations of the signaling circuitries caused by reprogrammed signaling networks as a consequence of targeted therapies, proteomics and kinome profiling can provide alternative, though technically challenging, omics approaches.

Mass spectrometry-based phosphoproteomics is an unbiased approach to studying the phosphorylation events of proteins, and can provide detailed views of the activation status of various signaling nodes within cells. In contrast, kinome profiling uses multiple kinase inhibitors that, arranged in a top-down fashion inside a column, present varying specificities to protein substrates [265]. Endogenous protein kinases that bind to these inhibitors are captured and analyzed by mass spectrometry to provide a global kinome representation. Phosphoproteomics-based methods have also been used to determine the activation states of kinases [266] and to establish new kinase–phosphosite relationships [267]. Comprehensive use of proteomics approaches for cancer therapeutics can be found in specialized reviews [268,269].

An understanding of how signaling networks adapt to perturbations induced by drug inhibitors, and how kinase–phosphosite relationships contribute to generate adapted signaling networks, can help design new combination therapies for cancer treatment.

Phosphoproteomics analyses have identified the CDK7/POLR2A axis as a new molecular node in patient-derived epithelial ovarian cancer (EOC), and it was shown that using the CDK7 inhibitor TZH1 suppressed the proliferation of EOC [270]. Furthermore, Hijazi and colleagues used chemical phosphoproteomics to reveal that while *PIK3CA* wild-type cells seem to depend on MAPK pathway activation, *PIK3CA* mutant cells more predominantly rely on AKT and mTOR signaling outputs for survival [271]. Moreover, phosphoproteomics analysis identified the protein kinase A (PKA)-regulated signaling network as pro-tumorigenic circuitry in G-protein α subunit (GNAS)-mutated small cell lung cancer cells [272]. Through phosphoproteomics, we recently found that the loss of PTEN protein phosphatase activity leads to an enrichment of phospho-peptides regulated by the glucocorticoid receptor, GR, and confirmed that GR activation sensitizes mutant PTEN cells to death [139].

Kinome profiling can also be used to identify mechanisms associated with cancer drug resistance. Lee et al. showed that the phosphorylation of Y397 and Y576 on the focal adhesion kinase (FAK) is associated with resistance to docetaxel in a metastatic prostate cancer cell line, and that a combination therapy with FAK tyrosine kinase inhibitor can overcome the resistance [273]. Moreover, it has been demonstrated that AURORA kinase A, AURKA, sustains mTOR phosphorylation levels in *PIK3CA*-mutated breast cancer cells treated with a pan-PI3K inhibitor or the AKT inhibitor MK-2206 [274]. AURKA inhibitors (e.g., MLN8237) were found to synergize with PI3K inhibitors to kill breast cancer cell lines in vitro and in xenograft models [274].

### 4.2. Functional Genomics for Cancer Therapy

Through functional genomics approaches, the cancer dependency map (DepMap) initiative (Broad Institute of MIT and Harvard) has used RNA interference (RNAi) and CRISPR/Cas9 loss-of-function gene-editing strategies to screen thousands of cancer cell lines and identify genes whose expression was found to be essential for cancer cell survival [275,276,277]. By using genetic information (e.g., mutation, copy number variation, expression, and methylation profiles) from the cancer cell line encyclopedia (CCLE), scientists can now predict novel synthetic lethality and identify new molecular markers whose targeting can kill cancer cells harboring specific genetic mutations. As an example, inhibition of Egl nine homolog 1 (EGLN1) through the pan-EGLN1 inhibitor FG-4592 was found to selectively reduce the viability of HIF1A-high [278] or ARID1A-mutant [279] human ovarian cancer cell lines. Moreover, targeting of the Werner syndrome ATP-dependent helicase (WRN) was shown to effectively suppress the growth of microsatellite instable (MSI) colorectal and endometrial carcinoma cell lines [280].

The DepMap initiative also supports the repurposing of non-oncologic drugs by performing chemical perturbation viability screening [281]. For instance, the alcohol dependence drug disulfiram was found to kill cancer cells with a reduced expression of metallothioneins [282]. In a nutshell, this effort could save costs and time required for de novo drug developments.

### 4.3. Event-Driven Pharmacology: A Novel Approach to Inhibit Cancer-Associated Proteins

Occupancy-driven pharmacology is the mainstream drug discovery paradigm for cancer treatment [283]. It relies on the use of small molecule inhibitors that occupy the functional-binding site of a protein and can disrupt protein interactions and their functions. Although small molecule inhibitors have been used in various cancer treatments, high doses of drug exposure are often required to maintain sufficient site occupancy in vivo. In turn, this can lead to off-target effects and unwanted toxicity. In contrast, event-driven pharmacology aims to target cancer-associated proteins, e.g., overexpressed or mutant proteins, and direct them to the ubiquitin–proteasome system (UPS) for elimination. Ubiquitination is a process by which misfolded or unfolded proteins are tagged with a chain of ubiquitin molecules to induce their degradation by the proteasome.

Proteolysis targeting chimeras (PROTACS) are bifunctional molecules containing a ligand-binding moiety linked to an E3 ligase-binding moiety [283]. The ligand-binding moiety recognizes the protein of interest (POI) and brings it to the proximity of an E3 ligase to form a ternary complex. The ternary complex then allows for the ubiquitination and elimination of the POI while the PROTAC is recycled ready for the next round of action.

Recently, PROTACS have been shown to outperform occupancy-driven small molecule inhibitors for multiple reasons [284]. First, PROTACS can recognize lysine residues on the surface of any protein, including commonly undruggable proteins such as transcription factors and scaffolding proteins, which would not be suitable targets for binding to small molecule inhibitors. Second, because PROTACS lead to the degradation of the POI, this process affects the catalytic activity as well as the potential scaffolding function of a protein. Moreover, after dissociating from the POI, PROTACS can cycle through multiple rounds of action and provide better pharmacokinetic profiles. There are around 600 different E3 ligases predicted in humans [285], which act in a tissue-specific and/or disease-specific manner and can therefore contribute to minimizing treatment-induced side effects while increasing the accuracy of targeted proteins. In fact, a recent proteomics study has shown that the VHL-based small molecule PROTAC can degrade ERRα and RIPK2 in tumor xenografts with nanomolar concentrations and showed limited off-target degradation effects [286].

Multiple cancer-related PROTACS have been developed so far, and these include PROTACS directed at EGFR [287,288], CDK4/6 [289,290], ERK1/2 [291], and PI3K [292]. Notably, Hines and colleagues designed a phosphoPROTACS ^ErbB2^PP_PI3K_ directed at PI3K [293]. The ^ErbB2^PP_PI3K_ consisted of a short stretch of ErbB3 intracellular domain bound to the SH2 domain of p85. Authors found that upon ErbB2/ErbB3 dimerization, the RTK phosphorylate ^ErbB2^PP_PI3K_ recruits PI3K for degradation and suppresses AKT activation in MCF7 breast cancer cells. It was found that INY-03-041 was able to inhibit PI3K/AKT signaling more effectively and potently than the PI3K inhibitor GDC-0068 [294].

In addition, the E3 ligase cereblon (CRBN)-based PROTAC used palbociclib to selectively target CDK6 for degradation [295], suppressing both the kinase-dependent and independent activity of CDK6 [296]. Burslem et al. have also shown that lapatinib-coupled PROTAC can target HER2 for degradation, leading to more sustainable inhibition of HER3, AKT, and ERK1/2 phosphorylation compared to non-functional diastereomers (stereoisomer with different spatial arrangements), which were unable to degrade HER2 [288]. Similarly, c-Met-targeted PROTAC can inhibit downstream HGF signaling for longer times than its diastereomer. Interestingly, this PROTAC also induced the degradation of the exon 14 splice mutant of c-Met, which was shown to be resistant to endogenous proteasomal degradation. This important study suggests that PROTACS may be more efficient in inhibiting oncogenic signals than small molecule inhibitors because PROTACS can delay and reduce the kinome rewiring, thereby preventing the reactivation of compensatory oncogenic pathways.

Although most of the reported PROTACS have been directed at druggable targets, extending this approach to difficult-to-target proteins is an achievable goal that could induce potent degradation of oncogenic proteins even in conditions of low protein affinity [283,297].

## 5. Conclusions

After more than five decades of intense studies, cancer still remains an incurable disease for too many patients. Tremendous progresses have been made in the treatment of many types of cancer, and important goals have been reached that improved patients’ outcomes. Although resistance to therapies and disease recurrence remain main hurdles for cancer treatment and cure, successes in the treatment of some cancers, such as acute promyelocytic leukemia (APL), should inspire the next generation of scientists to persevere in their search for better combination treatments and improved outcomes. As we have briefly discussed, event-driven pharmacology and PROTACS are just one example of new possible approaches that have the potential to revolutionize the cancer drug-discovery field, which can further rely on combinations with immunotherapies. Technological developments can provide new tools that further expand our understanding of cancer and also its adaptation to current therapies. This knowledge, coupled with preventive cancer screening programs and better combination treatments, remain our best chance to treat cancer.

## Data Availability

Not applicable.
